# Shoulder Function and Fatty Infiltration on MRI in Older Adults During Rehabilitation for Rotator Cuff Tear

**DOI:** 10.1093/geroni/igab046.3438

**Published:** 2021-12-17

**Authors:** Derik Davis, Ranyah Almardawi, Omer Awan, Lawrence Lo, Sagheer Ahmed, Shams Jubouri, Rao Gullapalli

**Affiliations:** 1 University of Maryland School of Medicine, Ellicott City, Maryland, United States; 2 University of Maryland School of Medicine, Baltimore, Maryland, United States; 3 Perelman School of Medicine at the University of Pennsylvania, Philadelphia, Pennsylvania, United States; 4 Geisel School of Medicine at Dartmouth, Lebanon, New Hampshire, United States

## Abstract

Rotator cuff tear is highly prevalent in older adults, with supraspinatus tendon tear (STT) the most common. Shoulder rehabilitation is a major treatment strategy, but supraspinatus-muscle-fatty infiltration (FI) and shoulder function in older adults with rotator cuff tear primarily managed by physical therapy (PT) is inadequately documented. We tested the hypothesis that older adults receiving usual-care PT when stratified by supraspinatus tear-status differ in supraspinatus FI [by quantitative Dixon fat fraction (FF) and semi-quantitative Goutallier grade (GG) on MRI] and shoulder function [by the American Shoulder and Elbow Surgeons score (ASES-score)] over time. Longitudinal cohort study (pilot): adults 60-85 years, PT-cohort (n=15) and control-cohort (n=25). Participants completed both shoulder MRI and ASES survey at baseline and follow-up visits. Kruskal-Wallis test compared within cohort among 3 groups: no tear (no-STT), partial-thickness tear (pt-STT), full-thickness tear (ft-STT). Mann-Whitney U test compared equivalent groups between cohorts. Baseline PT-cohort groups differed for GG (p=0.033) [no tear, 0.50±0.50;pt-STT, 1.11±0.22;ft-STT, 1.50±0.50] without difference in age, BMI, comorbidity, or ASES-score. Baseline control-cohort groups differed for FF (p=0.034) [no-tear, 5.77%±1.16%;pt-STT, 7.14%±6.26%;ft-STT, 21.44%±10.44%], without difference in age, BMI, comorbidity, or ASES-score. Baseline no-tear groups for ASES-score (p=0.049) differed between cohorts: PT-cohort (58.87±8.21) versus control-cohort (83.98±21.89). Both cohorts showed no difference in Δ-FF or Δ-GG over time. PT-cohort groups differed for Δ-ASES-score over time (p=0.042)[no-tear, 16.65±4.69;pt-STT, -7.24±0.94;ft-STT, 4.48±3.45], but control-cohort groups did not (p>0.050). Our results suggest differences exist for supraspinatus FI and self-reported shoulder function among older adults receiving PT for rotator cuff tear when stratified by supraspinatus tear-status.

